# A Haptoglobin Exon Copy Number Variant Associates With HIV-Associated Neurocognitive Impairment in European and African-Descent Populations

**DOI:** 10.3389/fgene.2021.756685

**Published:** 2021-12-22

**Authors:** Haimeng Bai, Harpreet Kaur, Asha R. Kallianpur, Todd Hulgan, Donald R. Franklin, Scott L. Letendre, Ronald J. Ellis, William S. Bush

**Affiliations:** ^1^ Department of Population and Quantitative Health Sciences, Cleveland Institute for Computational Biology, Case Western Reserve University, Cleveland, OH, United States; ^2^ Department of Nutrition, Case Western Reserve University School of Medicine, Cleveland, OH, United States; ^3^ Department of Genomic Medicine, Lerner Research Institute, Cleveland Clinic Foundation, Cleveland, OH, United States; ^4^ Department of Molecular Medicine, Case Western Reserve University School of Medicine, Cleveland, OH, United States; ^5^ Department of Medicine, Vanderbilt University School of Medicine, Nashville, TN, United States; ^6^ Department of Psychiatry, HIV Neurobehavioral Research Center, University of California, San Diego, San Diego, CA, United States; ^7^ Departments of Medicine and Psychiatry, University of California, San Diego, San Diego, CA, United States; ^8^ Departments of Neurosciences and Psychiatry, University of California, San Diego, San Diego, CA, United States

**Keywords:** haptoglobin, neurocognitive impairment, HIV, CHARTER, longitudinal, age, HIV-associated neurocognitive disorder, genetic imputation

## Abstract

A common two-exon deletion distinguishes the gene encoding the free hemoglobin capturing protein—haptoglobin (HP)–into two alleles: *HP1* and *HP2*. To evaluate the impact of this copy number variant (CNV) on neurocognitive impairment (NCI) in people living with HIV, we imputed this variant in 432 European-descent (EUR) and 491 African-descent (AFR) participants from the CNS HIV Antiretroviral Therapy Effects Research Study using an optimized imputation pipeline and evaluated its associations with NCI. At baseline, in AFR, the *HP2* allele decreased the odds of NCI (defined by a global deficit score, GDS, ⩾0.5 ; Odds Ratio, *OR* = 0.584, *p* = 0.022). However, in EUR, *HP2* increased the odds (*OR* = 2.081, *p* = 0.040) of NCI suggesting a detrimental effect. These effects were extended to longitudinal analyses using repeated measurements where the protective effect of the *HP2* allele in AFR became marginally significant (*p* = 0.054) and in EUR the detrimental effect increased in significance (*p* = 0.037). In EUR, the *HP2* allele slightly reduced the risk of NCI over time (*OR* = 0.028 per allele per year, *p* = 0.024). Further analyses of cognitive domain-specific impairment revealed that the *HP*-NCI effect was based on changes in learning, speed of information processing, and verbal domains over time differing by ancestry groups. Overall, these findings suggest that these functional *HP* CNV alleles influence the likelihood of NCI and contribute to changes in neurocognitive function over time in people living with HIV.

## 1 Introduction

Neurocognitive disorders have long been complications of HIV infection. Combination antiretroviral therapy (cART) has reduced morbidity and mortality in people living with HIV (PLWH) and remarkably prolonged their life expectancy (Grant et al., 2014; Watkins and Treisman, 2015). However, as PLWH age, neurologic complications like HIV-associated neurocognitive disorders (HAND) have become increasingly prevalent in both African-descent (AFR) and European-descent (EUR) populations (Antinori et al., 2007; Heaton et al., 2010; Grant et al., 2014). Ranked according to the severity of neurocognitive impairment (NCI), as established by Frascati criteria, the most severe yet uncommon form of HAND is HIV-associated dementia (HAD), followed by milder forms, including mild neurocognitive disorder (MND) and asymptomatic neurocognitive impairment (ANI) (Grant et al., 2014). Altogether, the overall prevalence of HAND is about 30–50% among randomly selected PLWH (Heaton et al., 2010; Jia et al., 2017). Typical phenotypes include mental slowing, memory loss, difficulties with complex tasks requiring executive function, and motor disorders (Simioni et al., 2010). Individuals also have behavioral abnormalities including apathy and a decrease in spontaneity or emotional responses (Simioni et al., 2010). NCI is the defining feature of HAND. It affects one or more of seven cognitive function domains, including verbal fluency, speed of information processing (SIP), learning, memory, motor function, attention and working memory, and executive function (Woods et al., 2004). The Global Deficit Score (GDS) represents an overall measure of neurocognitive performance based on a comprehensive neuropsychological test battery, and is widely used to rate HIV-associated NCI (Blackstone et al., 2012). Ranging from zero (unimpaired) to five (maximum severity) the GDS has been shown to be able to detect milder, HIV-associated cognitive impairment across multiple domains (Blackstone et al., 2012). It was previously shown that defining NCI as GDS ⩾0.5 yields the optimal balance between sensitivity and specificity (Blackstone et al., 2012).

HIV infection and its induced chronic neuroinflammation are key factors found to contribute towards the development of HIV-associated NCI. HIV virus infects the CNS within days of acute infection, causes neuropathological changes in the basal ganglia and the white matter, and leads to high rates of delirium, depression, opportunistic CNS infections, and dementia; long-term HIV replication occurs in astrocytes and microglia and compromises neuronal function (Simioni et al., 2010; Lutgen et al., 2020; Valdebenito et al., 2021). Elevated markers of immune activation and inflammation are commonly detected in cerebrospinal fluid (CSF) from PLWH who have HAND (Gannon et al., 2011). Infected monocytes crossing the blood-brain barrier (BBB) can infect perivascular and other microglia in the brain, a process which is further enhanced by inflammatory mediators released by these cells (Strazza et al., 2011; Saylor et al., 2016). Increases in activated monocytes in the peripheral blood also have been shown to be associated with HAND (Strazza et al., 2011). Moreover, the BBB is disrupted by HIV infection both at the beginning of the infection and after virus entry into the CNS through infected monocyte-macrophages and this may lead to generation of reactive oxygen species (ROS) in CNS and damage brain tissues (Strazza et al., 2011). Other established risk factors for NCI in PLWH include age, nadir CD4+ T cell count, anemia, possibly female sex and comorbidities (e.g., cardiometabolic disorders, substance abuse, hepatitis C) (Ellis et al., 2011; Nightingale et al., 2014; Watkins and Treisman, 2015; Kallianpur et al., 2016; Rubin and Maki, 2019). Furthermore, genetic studies have shown that host genetic variations also play an important role in NCI and its progression (Kallianpur and Levine, 2014; Jia et al., 2017; Olivier et al., 2018).

The plasma glycoprotein Haptoglobin (HP, with Ensembl ID: ENSG00000257017 and Entrez Gene ID: 3240) has potential functional relationships with NCI. HP is mostly generated in the liver and secreted into the blood where its major function is to bind free hemoglobin (Hb), a highly reactive oxygen carrier molecule, and facilitate its clearance (Schaer and Alayash, 2010; Ratanasopa et al., 2013; MacKellar and Vigerust, 2016). In the brain, oligodendrocytes can also synthesize HP, although HP is not synthesized in the brain under normal conditions (Zhao et al., 2009; Bulters et al., 2018). Higher CSF HP levels were found to be associated with NCI and HAND in PLWH who had minimal comorbidity from a study including both AFR and EUR populations (Kallianpur et al., 2019). HP has also been associated with the pathogenesis of other neurocognitive diseases, such as Alzheimer’s disease (AD) (Yerbury et al., 2009; Spagnuolo et al., 2014; Song et al., 2015; MacKellar and Vigerust, 2016). In addition, HP reduces the oxidation of apolipoprotein E (APOE), rendering APOE more soluble and better able to clear plasma lipids, thereby promoting its function in cholesterol homeostasis (Salvatore et al., 2009; Spagnuolo et al., 2014).

A common copy number variation (CNV) that spans 2 tandem exons of the *HP* gene distinguishes alleles *HP1* (one copy of exons 3 and 4) and *HP2* (two copies of exons 3 and 4) in humans (Boettger et al., 2016). Boettger *et al.* hypothesized in their work that the *HP2* allele is ancestral (based on comparisons with Neanderthal and Denisova genomes), and that the *HP1* allele arose due to multiple recurrent deletions across different human populations. The evolution of these alleles is also thought to follow different tracks in both AFR and EUR populations due to migration and potential natural selection (Boettger et al., 2016). This variant is not detected by typical genotyping methods but can be inferred from a group of single nucleotide polymorphisms (SNPs) within the *HP* gene region with high accuracy using genotype imputation (*r* ^2^ = 0.94 for EUR and *r* ^2^ = 0.92 for AFR) (Boettger et al., 2016). The *HP* CNV affects HP protein structure and function. Western blot experiments and electronic microscopy images have shown that the HP1-1 (both alleles are *HP1*, i.e., only HP1 protein is available) only forms a functional dimer, while the HP1-2 and HP2-2 can form multimers with linear and circular conformations (Bulters et al., 2018). Although all forms have similar Hb binding affinity, compared to HP1-1, the HP2-2 proteins have larger sizes, which lower binding capacity and result in lower efficiency in clearing Hb, thereby reducing protection against free-Hb-mediated oxidative damage (Melamed-Frank et al., 2001; MacKellar and Vigerust, 2016). The *HP* CNV was also reported to be associated with HIV outcomes: Caucasian PLWH with HP2-2 had a higher mortality rate, with a reduction in median survival of approximately 4 years, compared to PLWH with HP1-1 and HP1-2 (Delanghe et al., 1998). EUR PLWH who have HP2-2 also have higher HIV viral load (Delanghe et al., 1998; MacKellar and Vigerust, 2016).

It remains unclear whether the *HP* CNV is associated with NCI in PLWH, including individuals receiving suppressive cART. We address this question by imputing *HP* genotypes for the AFR and EUR participants in the CNS HIV Antiretroviral Therapy Effects Research (CHARTER) Study, a large, observational HIV cohort with comprehensive neurocognitive assessments and previously measured CSF HP protein levels, and evaluating the associations between *HP* genotype and NCI at baseline and over time.

## 2 Methods

### 2.1 CHARTER Study Population and Neurocognitive Assessments

The CHARTER Study is a prospective, observational study of neurocognitive outcomes in PLWH. Ambulatory, PLWH were enrolled at six medical centers in the U.S., as described previously (Heaton et al., 2010). Detailed, structured interviews and comprehensive neurocognitive examinations, as well as laboratory assessments were conducted to collect information on HIV disease and treatment-related factors from participants at baseline and 6-months follow-up visits according to a protocol that was standardized across sites. Details of CHARTER study eligibility and assessment protocols have been published before (Heaton et al., 2010). For participants who consented, CSF samples were also obtained by lumbar puncture. To assess the GDS, participants underwent a comprehensive test battery that involved seven neurocognitive domains and were assigned test scores which were then converted to demographically corrected standard scores (T-scores) (Heaton et al., 2010). A single T-score was calculated for each of the cognitive domains by averaging the T-scores for each of the tests in that domain. The domain impairment is determined when an individual’s T-score is below one standard deviation from the mean (Antinori et al., 2007). A composite GDS was then derived, as a continuous measurement, with deficit scores converted from standard domain T-scores, using a published objective algorithm (Carey et al., 2004; Heaton et al., 2010). Participants’ NCI status was also determined by applying a GDS cutoff of 0.5, with ‘Normal/Not impaired’ defined by a GDS <0.5 and ‘Impaired’ by a GDS ⩾0.5 (Carey et al., 2004; Jia et al., 2017). The GDS incorporated adjustments for practice (or learning) effects to account for prior neurocognitive testing. Neuro-relevant comorbid conditions were evaluated by experienced clinicians. Conditions such as developmental learning disability and major head injury with loss of consciousness were excluded and the rest were categorized as either “incidental” (absent, minimal and non-contributory) or “contributing” (mild-to-moderate) to NCI (Salvatore et al., 2009; Heaton et al., 2010).

### 2.2 Genotyping, Measurements of CSF HP Levels, and Known Factors Influencing NCI

Plasma HIV RNA (viral load) was determined by reverse transcriptase PCR (Heaton et al., 2010). The CD4+ nadir was obtained by self-report and confirmed by documented prior measurements in a subset of CHARTER Study participants (Ellis et al., 2011). CSF HP protein levels were quantified in 405 participants using multiplex bead-based suspension array immunoassays (Kallianpur et al., 2019). Detailed methods for quantification of CSF HP and other markers were published previously (Heaton et al., 2010; Ellis et al., 2011; Kallianpur et al., 2019). Genomic DNA was extracted from peripheral blood mononuclear cells collected at the baseline CHARTER visit using PUREGENE (GentraSystems, Inc., Minneapolis, MN). Genotyping was conducted using the Affymetrix Genome-Wide Human SNP Array 6.0^*TM*^ by the Vanderbilt Technologies for Advanced Genomics (VANTAGE) at Vanderbilt University in two batches: *n* = 576 samples were genotyped before 2009 and *n* = 506 (six repeated for QC) were genotyped in 2012, due to funding reasons only (Jia et al., 2017). Nevertheless, only minor changes were observed from explicit testing of batch effects (Jia et al., 2017). Due to limited accuracy for imputing *APOE*, the *APOE* genotypes were determined for a subset (*n* = 401) of CHARTER participants by genotyping of rs7412 and rs429358, using TaqMan predesigned SNP genotyping assays (C_904973_10 and C_30846793_20; Applied Biosystems, Foster City, CA) as described in a previous publication (Morgan et al., 2013).

### 2.3 Quality Control

The QC and basic data cleaning pipeline used for CHARTER genomic data was published previously (Jia et al., 2017); here we describe the additional QC work that was conducted for *HP* imputation. Since the HP reference panels are separated for AFR and EUR, we performed all the additional QC steps respectively for AFR and EUR participants. The overall genotyping call rate was checked for each study participant and we found the overall call rate was >95% for all samples. The whole dataset was then separated into the AFR set and the EUR set, according to ethnicity clusters defined by principal component (PC) ancestry clustering, which, as previously performed by the CHARTER study groups (Samuels et al., 2016; Jia et al., 2017), is an approach of assigning individuals’ ancestry groups by clustering of the continental-ancestry-corresponding PCs obtained from their genomic composition. In each ancestry group, for each SNP/marker, the overall call rate was then checked, respectively. Markers with <97% call rate (i.e., ⩾3% missing rate) within each population were pruned from the dataset. Finally, SNPs that failed the Hardy-Weinberg Equilibrium test (with *p*-value ⩾0.001 ) within each population were removed. The entire QC process was conducted using PLINKv1.9 (Chang et al., 2015; Purcell and Chang, 2019).

### 2.4 HP Genotype Imputation

We adopted published *HP* CNV imputation references for AFR and EUR (Boettger et al., 2016). An *HP* marker that was collapsed from all four *HP* subtype markers was added to the imputation reference with “0” represents *HP1* and “1” represents *HP2*. We performed *in silico* validation and found the *HP* genotype we obtained from the *HP* marker 100*%* identical to the genotype we obtained from subtype markers. The full lengths of chromosome 16 of the individuals in the *HP* imputation reference were extracted from 1,000 Genomes (1KG) data and pre-phased using SHAPEITv2 (Delaneau et al., 2013) software. The *HP* imputation markers were then extracted from the pre-phased chromosome 16 to obtain the phased *HP* imputation reference panels. Further validation of this imputation strategy and optimization of the IMPUTEv2 (Howie et al., 2009) software settings were conducted using: 1. *HP*-genotype-removed imputation reference as data input with an accuracy metric and 2. the GTEx sequencing and expression data by comparing the imputed *HP* genotype and the read count of the exon 4 & 5 junction (unique to *HP2*) from RNA sequencing. The *HP* region of all CHARTER Study participants was imputed for *HP* genotypes, using both of the phased references and samples that were extracted based on the reference population; i.e., EUR participants were extracted from the imputation using European reference and AFR participants were extracted from the imputation using African reference. The *HP* imputation was conducted using the IMPUTEv2 software.

### 2.5 Association Analyses

The imputed dosages were hardcalled using a 0.9 threshold; in other words, dosages ⩾0.9 were converted to genotypes and dosages <0.9 were replaced by ‘Null’ and excluded from hardcall analyses. The hardcalling and data preparation steps were conducted using Python3.7 programming language. Due to variations in *HP* allele frequency and other genetic complexities, analyses were conducted respectively for AFR and EUR. For continuous GDS as an outcome, analysis of variance (ANOVA) was applied, whereas for GDS defined NCI as the outcome, the *χ* ^2^-square test was used. Multivariate linear regression and logistic regression were used for continuous and categorical outcomes, respectively, with adjustments of age (continuous), CD4+ nadir (continuous), plasma HIV RNA (continuous), sex (categorical), comorbid condition (contributing vs. incidental), and CSF HP protein levels (continuous) if specified. Combined analyses of both populations were adjusted additionally for the first 3 PCs. Longitudinal analyses were conducted on repeated measurements of GDS, NCI, cognitive domain T-scores, and domain impairments with generalized estimating equations (GEE) method using age as the time variable. To obtain a more detailed estimation, we first estimated the ages of the records with a minimum increment of 0.5, and then fit into a GEE model with an empirical estimator and first-order auto-regressive (AR1) covariance structure to test if the *HP* CNV impacts the trajectory of changes of the outcomes. An identity link was used for the continuous outcomes and a logit link was used for the dichotomous outcomes. Moderation effects between variables were tested using interaction terms in models. In other words, a significant A-B interaction effect suggests that the effect of A on the outcome depends on B and vice versa. Additive and dominant genetic effects were tested for genetic variables by applying different coding methods. All hardcall analyses were performed using R statistical language. Specifically, the *geepack* (Yan, 2002; Yan and Fine, 2004; Halekoh et al., 2006) R library was used for longitudinal data analyses. SNPTEST (Marchini et al., 2007) software was used for frequentist analyses on continuous GDS and NCI using directly imputed dosages/certainties.

## 3 Results

### 3.1 Haptoglobin *HP2* Allele Associates With Lower CSF HP Levels

Statistics of all the study variables are in Table 1. Due to the complex evolutionary history of *HP* alleles in different populations, all analyses were conducted in AFR and EUR ancestral groups separately as well as jointly. The *HP* imputation process was validated by comparing the imputed *HP* genotypes and exon 4 & 5 junction counts from the Genotype-Tissue Expression (GTEx) RNA sequencing data (Supplementary Figure S1). *HP* genotypes were then imputed in the CHARTER AFR and EUR participants with high certainty (IMPUTEv2 info metric = 0.831 for AFR, and 0.830 for EUR). A low imputation dosage indicates a low certainty of the imputed genotype, thus, we filtered out the low confidence genotypes (hardcalls) with a stringent threshold, 0.9, to ensure that we had high quality genotypes for further analyses (Supplementary Figure S2). We obtained 371 hardcalls (86*%* of 432 samples) and 395 hardcalls (80*%* of 491 samples) in EUR and AFR, respectively. After hardcalling, Hardy-Weinberg Equilibrium was tested in both populations and no significant deviations were found.

**TABLE 1:** Summary of study variables.

			Baseline		Longitudinal	
Type			AFR (n = 395)	EUR (n = 371)	AFR (n = 393, Rec. = 1,358)	EUR (n = 371, Rec. = 1,461)
Factor	NCI	Normal	277	244	1314	1114
		Impaired	118	127	475	627
	Sex	Male	260	326	1273	1549
		Female	135	45	516	193
	Comorbidity	Minimal/No	236	258	1142	1231
		Mild-Moderate	159	113	647	511
	Memory Domain Impairment	Normal	267	279	1408	1213
		Impaired	128	92	381	528
	Learning Domain Impairment	Normal	260	239	1331	1202
		Impaired	135	132	458	539
	Verbal Domain Impairment	Normal	355	297	1574	1462
		Impaired	40	74	214	279
	Motor Domain Impairment	Normal	341	260	1451	1181
		Impaired	53	108	328	548
	SIP Domain Impairment	Normal	351	311	1602	1447
		Impaired	44	60	187	294

Numeric	Mean (SD)	IQR	Mean (SD)	IQR	Mean (SD)	IQR	Mean (SD)	IQR
GDS	0.392 (0.387)	0.529	0.506 (0.579)	0.533	0.373 (0.426)	0.466	0.494 (0.562)	0.600
Age	43.549 (8.029)	10	43.447 (9.302)	11	45.568 (7.765)	10	46.957 (9.471)	12
*Log*_10_(Plasma HIV RNA)	2.939 (1.295)	2.344	2.820 (1.313)	2.353	2.692 (1.262)	2.076	2.407 (1.134)	1.092
CD4+ Nadir (Cells/*μ*L)	198.987 (182.701)	254	238.515 (208.552)	249	169.060 (161.813)	242	207.146 (174.839)	240.5
Memory Domain T-score	45.577 (8.760)	12	47.232 (8.785)	10	47.664 (8.885)	12.5	45.908 (9.678)	13
Learning Domain T-score	43.954 (8.222)	11.75	42.849 (8.222)	11.75	46.317 (8.845)	12.5	45.286 (9.521)	13
Verbal Domain T-score	51.419 (8.653)	10.5	47.602 (8.495)	11.5	50.804 (8.678)	11	48.736 (8.946)	11.5
Motor Domain T-score	48.155 (9.382)	10	45.340 (11.017)	15.25	47.281 (10.131)	13	44.225 (10.624)	14.5
SIP Domain T-score	50.191 (7.754)	10.667	49.399 (9.484)	13.333	50.761 (8.533)	10	49.593 (9.874)	13.667

Showing hardcalled individuals only. The number in each cell shows the count for factor variables and the value for numeric variables. “SD” = standard deviation, “IQR” = interquartile range, “Rec.” = the number of records, and “SIP” = speed of information processing.

**FIGURE 1:**
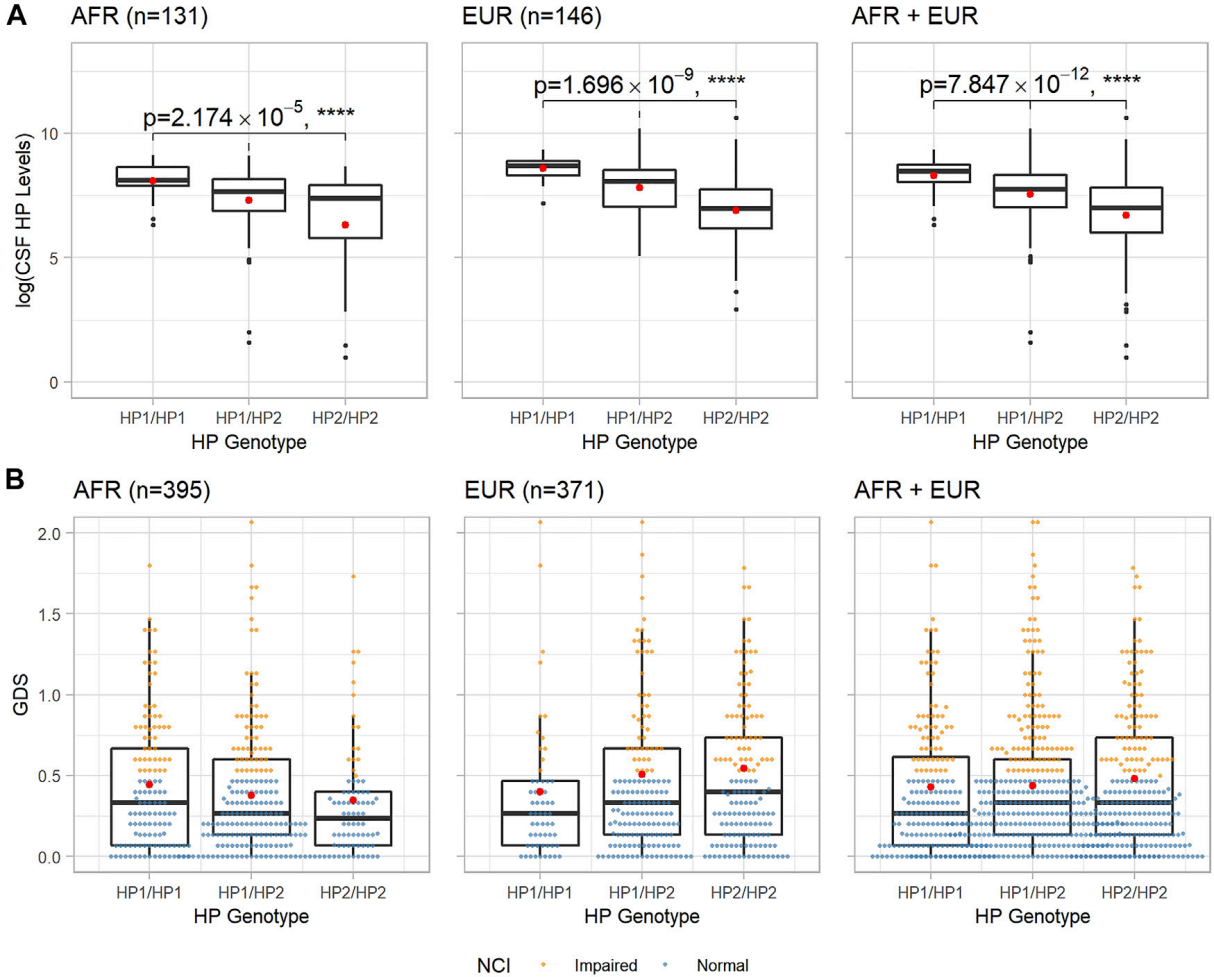
Boxplots of CSF HP protein levels and baseline GDS of each *HP* genotype in AFR and EUR ancestry groups. **(A)**: CSF HP protein levels in logarithmic scale vs. *HP* genotypes. *p*_values and asterisks indicating level of significance are from the ANOVA F-test. **(B)**: GDS at baseline vs. *HP* genotypes. In both panels, within each box, the black horizontal bar indicates the median value, and the red dot shows the mean value.

Associations between the *HP* CNV and CSF HP protein levels were determined using an ANOVA F-test among the participants who had CSF HP levels measured (*n* = 283, after hardcalling) (Kallianpur et al., 2019). In both populations, the presence of additional *HP2* alleles decreased the CSF HP protein levels, as shown in Figure 1A, (*p* = 2.17 × 10^–5^ in *n* = 131 AFR participants, *p* = 1.70 × 10^–9^ in *n* = 146 EUR participants, and *p* = 7.85 × 10^–12^ when combined).

### 3.2 Haptoglobin CNV Associates With Baseline NCI Differently Across Ancestry Groups

The distributions of baseline GDS for each *HP* genotype in AFR and EUR populations are shown in Figure 1B; we observed an almost linear relationship between the mean GDS of different *HP* genotypes and the number of *HP2* alleles in both AFR and EUR. However, neither ANOVA nor linear regression showed a statistically significant association between the *HP* CNV and the GDS. We then looked at the NCI as a dichotomous trait, defined by GDS ⩾0.5 as previously described (Heaton et al., 2010). *χ* ^2^-square tests showed a significant relationship between *HP* CNV and NCI in both populations, with *p* = 0.027 in AFR and *p* = 0.013 in EUR.

We adjusted for known risk factors for the dichotomous NCI in PLWH, including age, sex, comorbidity conditions, plasma HIV RNA (viral load), and CD4+ nadir in a logistic regression model as covariates (Heaton et al., 2010). In contrast to the consistent effects on CSF HP levels seen across both ancestry groups, we observed opposite directions of effect of the *HP2* allele on NCI in AFR and EUR individuals. As shown in Table 2, in 395 AFR participants, the *HP2* allele was associated with decreased odds of NCI (Odds Ratio, *OR* = 0.584, *p* = 0.022), while in 371 EUR participants, the *HP2* allele was associated with increased odds of NCI (*OR* = 2.081, *p* = 0.040).

**TABLE 2:** Baseline *HP2* dominant effect on NCI in the AFR and EUR participants.

	AFR (*n* = 395)			EUR (*n* = 371)		
Variable	OR	95% CI	p_value	OR	95% CI	p_value
Intercept	0.640	(0.273, 1.501)	0.600	0.312	(0.123, 0.792)	0.211
*HP2* Dominant	0.584	(0.462, 0.739)	**0.022**	2.081	(1.455, 2.977)	**0.040**
*Log*_10_(Plasma HIV RNA)	0.981	(0.898, 1.073)	0.830	1.023	(0.930, 1.126)	0.814
^a^Comorbidity: Mild-Moderate	1.994	(1.589, 2.502)	**0.002**	3.425	(2.680, 4.375)	**4.9 × 10^–7^**
Age (Month)	0.989	(0.974, 1.004)	0.460	0.99	(0.976, 1.004)	0.486
Sex: Female	1.188	(0.936, 1.507)	0.471	1.379	(0.974, 1.95)	0.356
CD4+ Nadir (Cells/*μ*L)	1.000	(0.999, 1.000)	0.635	0.998	(0.997, 0.998)	**1.43 × 10^–3^**

The OR, and 95% confidence intervals (CI) are converted from effects estimated from a logistic regression model. Bold indicates statistical significant.

a Individuals with severe comorbidity were removed from analyses. Effect shows mild-to-moderate comorbidity compared to absent, minimal and non-contributory comorbidity.

To account for uncertainty in genotype imputation estimations, we also tested the association between NCI and *HP2* using directly imputed dosages rather than hardcalls. In EUR, we were able to detect a significant additive effect of *HP2* dosage with *OR* = 1.605, (*p* = 0.002), which is similar to what we obtained using hardcalls. No significant associations were found in AFR using directly imputed allele dosages.

We also found that the effect of *HP2* on NCI is independent from its association with CSF HP protein levels in both EUR and AFR PLWH. Although the sample size was limited, we performed sensitivity tests with the CSF HP protein levels to see if the effect of the *HP* CNV is driven by changes in the HP protein levels. Adjusting for the CSF HP levels did not change the significance of the *HP2* alleles in either EUR or AFR PLWH. From Supplementary Table S1, we could see that the CSF HP levels is not significantly associated with NCI risk in either AFR or EUR individuals. In fact, this adjustment strengthened the *HP* association in EUR (Supplementary Table S1).

### 3.3 The *HP* CNV Influences NCI Over Time

Given the longitudinal study design of CHARTER, we were also able to examine the effect of the *HP* CNV on NCI as the study participants age. For the AFR group, the median follow-up time is 6 months and the mean is 1.406 years. While in EUR, the median follow-up time is 6 months and the mean is 1.679 years.

**FIGURE 2:**
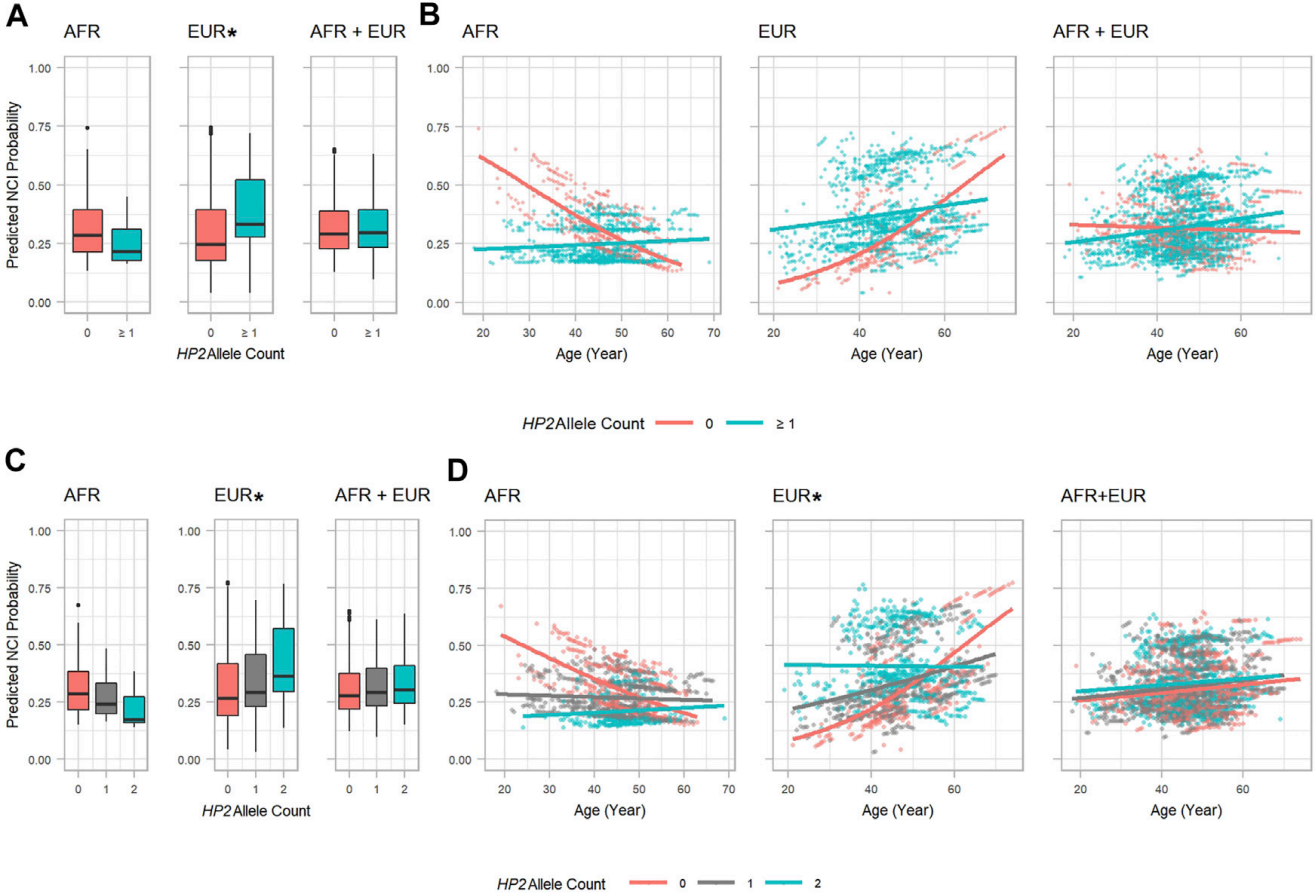
*HP2* Dominant Effect on Predicted NCI Probability in the AFR and EUR Participants. **(A, C)**: Boxplots showing the main effect of *HP2* on predicted NCI probabilities. **(B, D)**: Scatter plots with trendlines showing the effect of age on predicted NCI probabilities. Probabilities in **(A, B)** are from models shown in Table 3. Probabilities in **(C, D)** are from models shown in Table 4. Plots are colored by *HP2* status. Additional adjustment of the first 3 PCs was used in the AFR + EUR analysis. “∗” indicates statistical significance.

As shown in Table 3, EUR PLWH having *HP2* were at 10.276 higher OR (*p* = 0.037) of developing NCI than PLWH that do not have *HP2* (Figure 2A). This OR decreased by 0.961 per year (*p* = 0.077, Figure 2B). This decrease reflects a cumulative change in risk; for example, a decrease of 0.961 means the individuals probability of NCI equals the *probability of the previous year* × 0.961. We also noticed that though the risk of NCI for EUR *HP1/HP1* individuals was lower than *HP2* individuals at the beginning, it increased faster and became greater than *HP2* individuals after approximately 58 years of age. In contrast, though only marginally significant, AFR PLWH with *HP2* had lower risk (*OR* = 0.111, *p* = 0.054) of developing NCI compared to people without *HP2* (Figure 2A). The directions of the *HP2* effect on NCI were consistent with the baseline models within each population. These effects are visualized in Figure 2. We see a dramatic change in the trend of predicted NCI probability over age in both AFR and EUR between *HP2* dominant and *HP1/HP1* individuals. These effects were not observed from the analyses that combines the AFR and EUR participants as the opposing effects of the *HP* alleles in each population cancel each other out (Figure 2). We also detected an additive effect of *HP2* on NCI in EUR as shown in Table 4. In EUR, each copy of *HP2* allele was associated with a 4.719 increase in the OR (*p* = 0.009) of developing NCI (Figure 2C). This OR decreased by 0.972 per *HP2* allele per year (*p* = 0.024, Figure 2D). These effects are not statistically significant in the AFR or combined analyses (Table 4).

**TABLE 3:** *HP2* dominant effect on NCI in the AFR and EUR participants over time.

	AFR (*n* = 393, Rec. = 1,358)			EUR (*n* = 371, Rec. = 1,461)		
Variable	OR	95% CI	p_value	OR	95% CI	p_value
Intercept	1.743	(0.626, 4.851)	0.587	0.029	(0.011, 0.078)	**3.80 × 10** ^**–4**^
*HP2* Dominant	0.111	(0.035, 0.347)	0.054	10.276	(3.363, 31.404)	**0.037**
Age	0.960	(0.940, 0.981)	0.061	1.051	(1.031, 1.071)	**0.010**
Sex: Female	1.331	(1.067, 1.661)	0.196	1.122	(0.817, 1.541)	0.716
*Log*_10_(Plasma HIV RNA)	1.058	(0.996, 1.125)	0.350	1.112	(1.052, 1.176)	0.055
CD4+ Nadir (Cells/*μ*L)	1.000	(0.999, 1.001)	0.844	0.998	(0.998, 0.999)	**0.002**
^a^Comorbidity: Mild-Moderate	2.113	(1.718, 2.599)	**3.00 × 10** ^**–4**^	3.084	(2.496, 3.811)	**1.04 × 10** ^**–7**^
*HP2* Dominant × Age	1.042	(1.016, 1.068)	0.108	0.961	(0.939, 0.983)	0.077

The table shows the effects estimated from a GEE, empirical estimator. “Rec.” indicates the number of records. Bold indicates statistical significant.

a Individuals with severe comorbidity were removed from analyses. Effect shows mild-to-moderate comorbidity compared to absent, minimal and non-contributory comorbidity.

**TABLE 4:** *HP2* additive effect on NCI in the AFR and EUR participants over time.

	AFR (*n* = 393, Rec. = 1,358)			EUR (*n* = 371, Rec. = 1,461)		
Variable	OR	95% CI	p_value	OR	95% CI	p_value
Intercept	1.004	(0.395, 2.551)	0.996	0.029	(0.012, 0.070)	**7.46 × 10** ^**–5**^
*HP2* Additive	0.322	(0.138, 0.750)	0.180	4.719	(2.599, 8.567)	**0.009**
Age	0.972	(0.953, 0.990)	0.134	1.053	(1.034, 1.072)	**0.004**
Sex: Female	1.334	(1.072, 1.660)	0.188	1.151	(0.831, 1.594)	0.665
*Log*_10_(Plasma HIV RNA)	1.059	(0.997, 1.125)	0.344	1.113	(1.053, 1.176)	0.053
CD4+ Nadir (Cells/*μ*L)	1.000	(0.999, 1.000)	0.817	0.998	(0.998, 0.999)	**0.002**
^a^Comorbidity: Mild-Moderate	2.109	(1.715, 2.594)	**3.06 × 10** ^**–4**^	3.103	(2.510, 3.837)	**9.30 × 10** ^**–8**^
*HP2* Additive × Age	1.020	(1.001, 1.039)	0.290	0.972	(0.960, 0.984)	**0.024**

The table shows the effects estimated from a GEE, empirical estimator. “Rec.” indicates the number of records. Bold indicates statistical significant.

a Individuals with severe comorbidity were removed from analyses. Effect shows mild-to-moderate comorbidity compared to absent, minimal and non-contributory comorbidity.

HIV RNA levels were significantly different by *HP* genotype (Supplementary Figures S3–S6), suggesting that the risk for NCI may be influenced by altering viral load. To examine the potential mediation effect of the HIV RNA, we re-ran the analyses in the virus suppressed subgroup [⩽ lower limit of quantitation (LLQ)]. Despite changes in significance levels in some tests due to dramatic decreases in sample sizes, the directions of the effects remained consistent in the longitudinal analyses (Supplementary Tables S2, S3). Further analyses adjusting for the ⩽ LLQ status showed that the ⩽ LLQ status did not significantly contribute to NCI risk (Supplementary Tables S4, S5).

### *3.4 HP* CNV Associations to NCI Are Driven by Changes in Specific Domains

**FIGURE 3:**
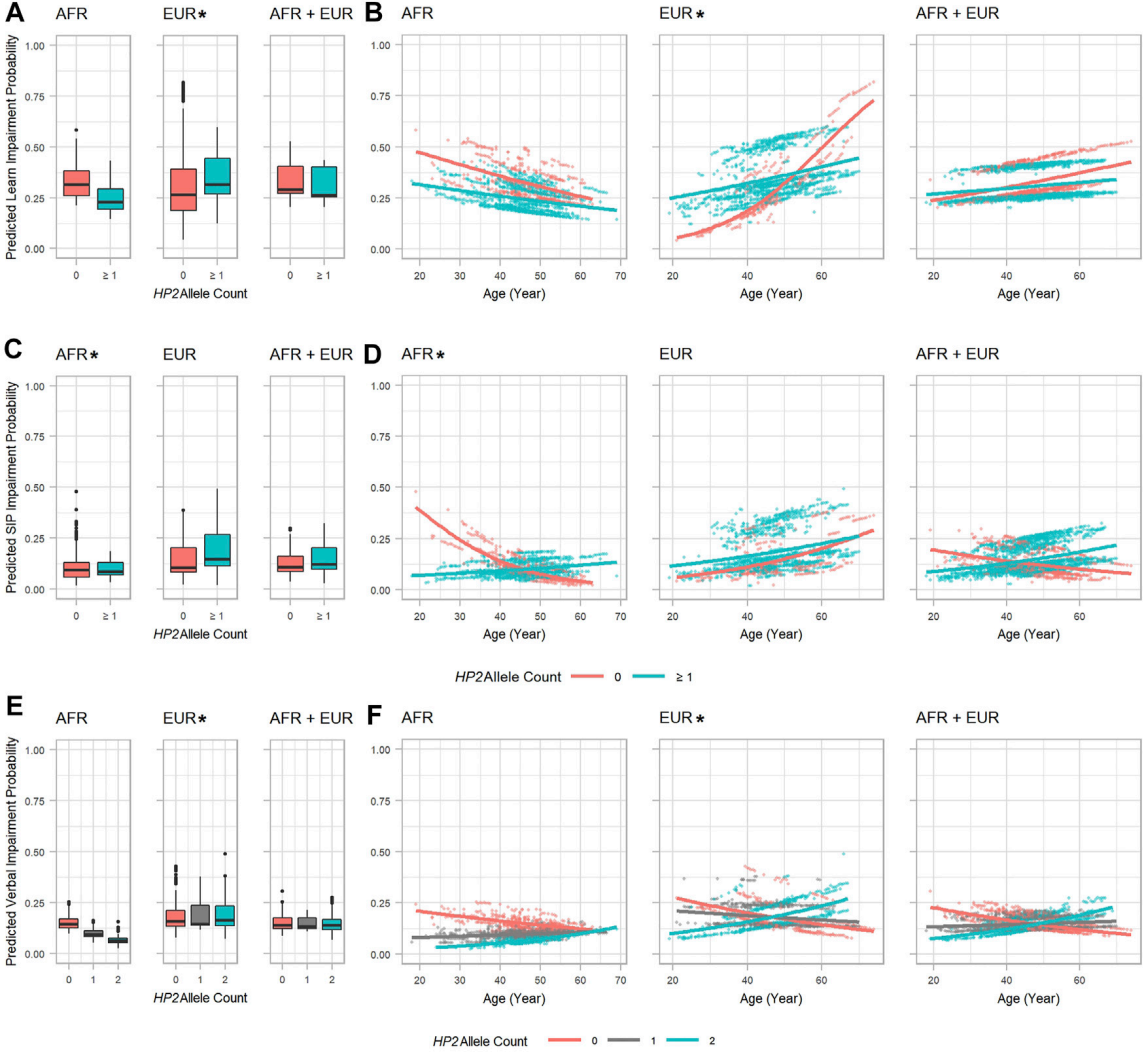
*HP2* effect on predicted specific cognitive domain impairment probability in the AFR and EUR participants. **(A, C)** and **(E)**: Boxplots showing the effect of *HP2* on predicted learning, SIP, and verbal domain impairment probabilities, respectively. **(B, D)**, and **(F)**: Scatter plots with trendlines showing the effect of age on predicted learning, SIP, and verbal domain impairment probabilities, respectively. **(A, B)** are from the model in Supplementary Table S6. **(C, D)** are from the model in Supplementary Table S8. **(E, F)** are from the model in Supplementary Table S7. Plots are colored by *HP2* status. Additional adjustment of the first 3 PCs was used in AFR + EUR analyses. “∗” indicates statistical significance.

We further decomposed the global model of NCI risk by investigating which cognitive domains are significantly affected by the *HP* CNV by conducting longitudinal analyses using domain impairments as outcomes. The higher risk of NCI in EUR with *HP2* is likely driven by an increased risk in the learning domain impairment. This effect is slightly (but significantly) offset by a significant decrease in risk for verbal domain impairment based on an additive model of the *HP* alleles. These two effects, combined with more modest (non-significant) effects in other domains together create an overall increase in risk for NCI (Figure 3, Supplementary Tables S6, S7). In EUR, PLWH with *HP2* had a higher OR of impairment in the learning domain (*OR* = 14.526, *p* = 0.019, Supplementary Table S6 and Figure 3A) with the risk reducing over time (by *OR* = 0.951, *p* = 0.030 per year of age, Supplementary Table S6). These over-time effects are reflected in Figure 3B where we observed a dramatic difference in slope between *HP2* dominant and *HP1* individuals. No statistically significant effects were noted in the AFR and combined (AFR + EUR) analyses (Figure 3B). We also found that with each *HP2* allele, EUR PLWH have a significant decrease in risk of verbal domain impairment (*OR* = 0.259, *p* = 0.019, Supplementary Table S7 and Figure 3E). This effect is smaller than the contrasting effect of *HP2* on the learning domain, and over time this risk modestly increases by *OR* = 1.020 per year (*p* = 0.023, Supplementary Table S7 and Figure 3F). In AFR, the *HP2* allele was associated with a lower risk of impairment in the speed of information processing domain (Figure 3 and Supplementary Table S8) with this risk increasing by *OR* = 1.072, per year (*p* = 0.038, Supplementary Table S8 and Figure 3D).

From Figure 3B, similar to the global NCI risk, we also observed an intersection point of the trend lines between 50 and 60 years of age, after which the risk of learning impairment for EUR *HP1/HP1* individuals became higher than *HP2* individuals. This alteration was also found in the verbal and speed of information processing domains. In both EUR and AFR, *HP* is associated with some additional changes to cognitive domain T-scores that did not impact impairment (see Supplementary Tables S9, S10).

## 4 Discussion

We report that a functional CNV in the *HP* gene is associated with NCI in PLWH. We found that both AFR and EUR individuals with more *HP2* alleles in our study sample had significantly lower HP levels. Despite the consistent direction of the relationship between CSF HP and *HP2* alleles in these two populations, however, the *HP* CNV showed differing directions of association with NCI at baseline in AFR and EUR populations. At baseline, having one or more copies of the *HP2* allele was protective in AFR, but detrimental in EUR participants. In longitudinal analyses, this effect became non-significant in AFR while it became more significant in EUR plus a significant over-time effect. Though not significant globally, in AFR, *HP* variation still impacted the speed of information processing cognitive domain. In EUR, the *HP2* allele affected NCI through the learning and verbal cognitive domains. The consistent association between CSF HP protein levels and the *HP2* variation in both the EUR and AFR groups provides additional support for our *HP* imputation process, and for some similarity in the basic biological impact of the alleles, even though their ultimate effects on cognitive impairment may differ.

Our finding of reduced cognitive decline among AFR individuals with HIV and *HP2* alleles is consistent with findings from a cohort study of 466 HIV-negative African-American adults with type 2 diabetes, which found that HP1-1 individuals had poorer cognitive function at baseline and smaller cognitive decline over time compared to other *HP* genotypes adjusting for key demographic and cardiovascular risk factors (Beeri et al., 2018). Our finding of elevated risk of NCI in EUR also, to some extent, agrees with the previous findings in 653 Caucasian PLWH that individuals with HP2-2 had a higher mortality rate and reduced median survival compared to HP1-1 and HP1-2 (Delanghe et al., 1998). The disparate effects between AFR and EUR individuals noted in this study is not uncommon. The evolution of the *HP* alleles have progressed differently in these populations, which have been exposed to very different selection pressures and disease risks, as is described by Boettger *et al.* and others (Gichohi-Wainaina et al., 2016; Rametta et al., 2020). With regard to the *HP* alleles, divergent HP1-1 associations with cognitive function have been reported to differ between Ashkenazi Jews and non-Ashkenazi Jews with type 2 diabetes (Gichohi-Wainaina et al., 2016). Thus, the opposing effects in the AFR and EUR populations are likely due to the interactions of these alleles with other genetic, environmental, and socio-cultural factors that influence the complex clinical trajectory of NCI in PLWH.

Haptoglobin plays an intricate role in iron homeostasis and the inflammatory response within the CNS (Bulters et al., 2018) which likely influences a variety of body functions across the lifespan. HP in the CSF can protect neurons from being damaged by the products of red blood cell lysis including Hb and iron-mediated ROS. HP may also assist in the stable formation of *β*-amyloid (A*β*) and APOE complex, thus, helping the clearance of A*β* (Spagnuolo et al., 2014). Prior work has shown HP2 has less efficient antioxidative activity relative to HP1. Since HP also serves as an antioxidant for APOE, *HP2* may lead to enhanced accumulation of A*β* and further the deterioration of the BBB (Montagne et al., 2020). Multiple biomarkers (such as A*β*42, tau, etc.) have been explored or used for clinical diagnosis of AD, and of these, S100*β* is a promising biomarker for BBB damage whose increase in serum indicates potential leakage of BBB (Marchi et al., 2003; Blennow and Zetterberg, 2018; Kadry et al., 2020). Future studies of these biomarkers in PLWH may reveal additional insights into the mechanism of *HP* CNV on NCI. On the other hand, HP2 may be more active in promoting tissue repair in chronic inflammatory conditions (Cid et al., 1993), which may have specific ramifications in the context of neuroinflammation in PLWH. Thus, over time, individuals with HP2 could display a symptomatic palliation of NCI.

As indicated by a wealth of neuroimaging and neurobehavioral studies, age is a strong risk factor for the development of neurocognitive decline and NCI among PLWH (Watkins and Treisman, 2015). In PLWH, accelerated aging was found and associated with HAND, a disorder with severe NCI (Levine et al., 2016). AD and Parkinson’s disease (PD) related pathological changes are also observed in ART-treated PLWH including elevated hyperphosphorylated tau protein in the hippocampus and A*β* deposition in the frontal cortex and hippocampus (Gannon et al., 2011). Thus, some researchers think HAND is associated with accelerated aging. Other researchers argue that HAND symptoms are induced by HIV infection and the use of antiretroviral therapy. Studies found that the HIV viral load is significantly higher in participants that develop NCI later yet age remains a risk modifier (Becker et al., 2004). Other mechanisms are also possible. However, given the current longevity of PLWH, we may not be able to fully understand the combined role of cART and HIV (Watkins and Treisman, 2015). Overall, NCI is a complex phenotype, and its relationship with *HP* is also complicated, and our associations show effects that are not easily delineated mechanistically.

To sum up on our findings, in addition to the existing effects of HIV viral load, HIV duration, and aging from previous publications (Ellis et al., 1997; Robertson et al., 1998; Becker et al., 2004; Simioni et al., 2010; Gannon et al., 2011; Watkins and Treisman, 2015; Olivier et al., 2018), the *HP* alleles are associated with NCI in PLWH, especially with an increased risk of NCI in EUR as well as a significantly alteration on the change of NCI risk from aging. Furthermore, it is unlikely that the effect of *HP2* on NCI is exclusively mediated by the HIV RNA because: 1. the analyses restricted to virally suppressed individuals illustrated that *HP* has an independent effect on NCI, 2. the *HP* association on NCI is robust after adjustment of HIV RNA. Given the known interaction of HP and APOE, an interaction effect of *HP* and *APOE* alleles may also exist. However, the nature of the CHARTER study data limited our analyses. Several key variables were collected only within different subgroups and the number of samples that have two or more of those variables available is even smaller. Thus, due to limited testing of *APOE* genotype status in CHARTER Study participants we were unable to draw a clear conclusion involving APOE as the sample size drops substantially for *APOE* genotype stratified analyses. We also tried to impute the CHARTER *APOE* status. However, the two SNPs required to infer *APOE* genotypes were imputed with low *r* ^2^ values: *r* ^2^ = 0.664 using the Haplotype Reference Consortium (HRC) reference and *r* ^2^ = 0.439 14 using the 1KG reference for rs429358, and *r* ^2^ = 0.658 using HRC and *r* ^2^ = 0.460 using 1KG for rs7412, and could not be used. Like the *APOE*, serum HP protein levels were also tested only within a limited subgroup that have *HP* hardcalls (*n* = 25 in AFR, and *n* = 48 in EUR), and we have limited power to assess the association between the *HP* genotype and serum HP protein levels. The overall genotyping call rate was >95% for all samples. The genotyping platform has limited our ability to impute the *HP* genotypes for some individuals with high confidence, though; we were still able to obtain *HP* genotypes in >80% of participants. Despite these limitations, we have identified a statistically significant, though biologically complex relationship between functional *HP* CNV alleles and the risk of NCI in PLWH as they age.

## Code Availability

Code for the analyses of this study could be found at: https://github.com/bushlab-genomics/Haptoglobin-CHARTER.

## Data Availability

The data analyzed in this study is subject to the following licenses/restrictions: With regards to access to the data, the authors cannot make the data publicly available as they have obtained it from a third party, the CHARTER group. Requests to access these datasets should be directed to https://nntc.org/content/requests.

## Abbreviations

Aβ, β-amyloid; AFR, African-descent; ANI, Asymptomatic neurocognitive impairment; APOE, apolipoprotein E; BBB, blood-brain barrier; AR1, first-order autoregressive; cART, Combination antiretroviral therapy; CNV, copy number variant; CNS, central nervous system; CSF, cerebrospinal fluid; CHARTER, CNS HIV antiretroviral therapy effects research; EUR, European-descent; GDS, global deficit score; GTEx, genotype-tissue expression; GEE, generalized estimating equations; HIV, human immunodeficiency virus; HP, haptoglobin; HRC, Haplotype reference consortium; HAND, HIV-associated neurocognitive disorders; HAD, HIV-associated dementia; LLQ, lower limit of quantitation; MND, mild neurocognitive disorder; NCI, neurocognitive impairment; OR, odds ratio; PLWH, people living with HIV; PC, principal components; QC, quality control; ROS, Reactive oxygen species; SIP, speed of information processing; 1KG, 1,000 Genomes.
