# Supplementary material for: A Haptoglobin Exon Copy Number Variant Associates With HIV-Associated Neurocognitive Impairment in European and African-Descent Populations
Source: Front Genet. 2021 Dec 22;12:756685. doi: 10.3389/fgene.2021.756685 (PMC8727522; doi:10.3389/fgene.2021.756685)
Supplement: Supplementary file 1 [file DataSheet1.pdf]

Supplementary Material

1 SUPPLEMENTARY TEXT

Adjustment of CSF HP Protein Levels at Baseline

The CSF HP protein levels were adjusted in the baseline NCI logistic regression model in log scale. From Table S1, we could see that the CSF HP levels is not significantly associated with NCI risk in either AFR or EUR individuals. *HP2* dominant effect become more significant in EUR but lost significance in AFR.

Sensitivity Tests on HIV Viral Load LLQ Subgroups

We sought to test if the *HP* effect on NCI is mediated by the HIV RNA level, and we conducted tests within the viral load suppressed subgroup [$\leqslant$ lower limit of quantitation (LLQ)]. The number of samples and records dropped drastically and so did the significance levels of the effects, but they remained in the same directions (Table S2 and S3. We then included both HIV RNA and LLQ status to see if the effect of HIV RNA could be cloaked by the LLQ status. From the results (Table S4 and S5), we could see that there is no evidence showing an effect of LLQ status on NCI for both plasma and CSF.

Longitudinal Effects of HP on Cognitive Domains

Longitudinal effects of *HP* on cognitive domain impairments are shown in Tables S6-S8. In AFR, verbal domain T-score is dominantly associated with *HP2* by 10.499 ± 4.942, ($p = 0.034$, Table S9). In addition, *HP2* is recessively associated with an increase of Memory domain T-score by 9.855 ± 4.995 ($p = 0.049$, Table S9). In EUR, *HP2* dominantly decreases the learning domain T-score by 10.956 ± 4.068, ($p = 0.007$, Table S10) while increases by 0.213 ± 0.088, ($p = 0.016$, Table S10) per year. *HP2* also dominantly decreases the memory domain T-score by -11.080 ± 4.079 ($p = 0.007$, Table S10) while increases by 0.212 ± 0.091, ($p = 0.020$, Table S10). Moreover, EUR PWH with *HP2* have a 11.850 ± 6.043 lower motor domain T-score than people without ($p = 0.050$, Table S10).

Other Effects of HP on NCI at Baseline

In AFR, an increasing number of *HP2* alleles was associated with decreased odds of NCI (Odds Ratio, $OR = 0.668$, $p = 0.015$, Table S11). In EUR, *HP2* additively increased the odds of NCI ($OR = 1.634$, $p = 0.004$, Table S11). In EUR, *HP1* also dominantly decreased the odds of NCI (Table S12).

Other Sensitivity Tests on HIV Viral Load

The associations with *HP* variation and GDS/NCI were tested for both plasma and CSF HIV RNA with various adjustments. No evidence shows a direct longitudinal association between the HIV RNA and the *HP* variation. Besides super strong association with the plasma HIV RNA as expected, the CSF HIV RNA also associates with the *HP* variation (Table S13). This effect is only in AFR not the EUR, that the *HP1* increases the CSF HIV RNA while the *HP2* decreases it additively (Table S13). In EUR, the *HP2* dominant and additive effects on NCI is robust for the adjustment of plasma or CSF HIV RNA with HIV duration (Table S14 and S15). In addition to the *HP2* dominant effect, plasma HIV RNA significantly associates with a higher risk of NCI after adjusting for an interaction of plasma HIV RNA and HIV duration (Table S14) and so does the CSF HIV RNA (Table S15).

2 SUPPLEMENTARY TABLES

Table S1. Baseline *HP2* dominant effect on NCI in the AFR and EUR participants with adjustment of CSF HP protein levels.

	AFR (n=170)			EUR (n=171)		
Variable	OR	95% CI	p_value	OR	95% CI	p_value
Intercept	112.373	(9.994, 1263.507)	0.051	0.697	(0.096, 5.055)	0.856
HP2 Dominant	1.001	(0.626, 1.600)	0.999	0.242	(0.127, 0.459)	**0.027**
Age	0.971	(0.943, 1.000)	0.323	1.001	(0.977, 1.026)	0.958
Sex: Female	0.730	(0.463, 1.153)	0.491	0.991	(0.549, 1.790)	0.988
Log_{10}(CSF HP protein)	0.727	(0.588, 0.898)	0.132	1.068	(0.909, 1.253)	0.683
Log_{10}(Plasma HIV RNA)	0.959	(0.809, 1.136)	0.805	1.084	(0.917, 1.280)	0.630
CD4+ Nadir (Cells/μL)	1.000	(0.998, 1.001)	0.696	1.002	(1.001, 1.004)	0.052
Comorbidity: Mild-Moderate	2.976	(1.958, 4.522)	**0.009**	4.838	(3.192, 7.334)	$\mathbf{1.507 \times 10^{-4}}$

Effect estimates are from a logistic regression model.

Table S2. *HP2* additive effect on NCI in the AFR and EUR plasma viral load suppressed subgroup over Time.

	AFR (n=192, Rec.=543)			EUR (n=202, Rec.=703)		
Variable	OR	95% CI	p_value	OR	95% CI	p_value
Intercept	1.109	(0.272, 4.519)	0.941	0.116	(0.036, 0.375)	0.067
HP2 Additive	0.220	(0.047, 1.038)	0.329	2.689	(1.156, 6.257)	0.241
Age	0.965	(0.938, 0.993)	0.217	1.031	(1.008, 1.056)	0.186
Sex: Female	1.357	(0.982, 1.877)	0.346	1.516	(1.005, 2.288)	0.312
CD4+ Nadir (Cells/μL)	1.000	(0.999, 1.001)	0.819	2.816	(2.124, 3.732)	$\mathbf{2.391 \times 10^{-4}}$
Comorbidity: Mild-Moderate	2.086	(1.546, 2.813)	**0.014**	0.998	(0.998, 0.999)	0.069
HP2 Additive × Age	1.031	(0.998, 1.066)	0.353	0.982	(0.966, 0.999)	0.288

Effects are estimated from a GEE empirical estimator.

Table S3. *HP2* additive effect on NCI in the AFR and EUR CSF viral load suppressed subgroup over time.

	AFR (n=247, Rec.=765)			EUR (n=261, Rec.=977)		
Variable	OR	95% CI	p_value	OR	95% CI	p_value
Intercept	1.700	(0.510, 5.664)	0.659	0.054	(0.020, 0.148)	**0.004**
HP2 Additive	0.150	(0.045, 0.500)	0.115	2.254	(1.101, 4.616)	0.257
Age	0.962	(0.938, 0.986)	0.119	1.046	(1.025, 1.068)	**0.028**
Sex: Female	1.173	(0.888, 1.550)	0.566	1.140	(0.775, 1.677)	0.734
CD4+ Nadir (Cells/μL)	1.000	(0.999, 1.001)	0.801	3.226	(2.528, 4.116)	$\mathbf{1.559 \times 10^{-6}}$
Comorbidity: Mild-Moderate	2.296	(1.779, 2.963)	**0.001**	0.999	(0.998, 0.999)	0.063
HP2 Additive × Age	1.039	(1.012, 1.066)	0.144	0.985	(0.971, 1.000)	0.301

Effects are estimated from a GEE empirical estimator.

Table S4. *HP2* additive effect on NCI in the AFR and EUR participants with adjustment of plasma LLQ status over time.

	AFR (n=393, Rec.=1,358)			EUR (n=371, Rec.=1,461)		
Variable	OR	95% CI	*p*_value	OR	95% CI	*p*_value
Intercept	1.120	(0.452, 2.772)	0.901	0.045	(0.018, 0.108)	$\mathbf{4.730 \times 10^{-4}}$
HP2 Additive	0.321	(0.137, 0.750)	0.181	4.469	(2.451, 8.148)	**0.013**
Age	0.970	(0.952, 0.989)	0.117	1.049	(1.030, 1.068)	**0.009**
>LLQ	1.196	(1.046, 1.368)	0.181	1.010	(0.906, 1.125)	0.929
Sex: Female	1.325	(1.066, 1.648)	0.197	1.159	(0.842, 1.596)	0.644
CD4+ Nadir (Cells/μL)	1.000	(0.999, 1.000)	0.778	0.998	(0.998, 0.999)	**0.003**
Comorbidity: Mild-Moderate	2.121	(1.724, 2.609)	$\mathbf{2.847 \times 10^{-4}}$	3.091	(2.504, 3.816)	$\mathbf{8.319 \times 10^{-8}}$
HP2 Additive × Age	1.020	(1.001, 1.039)	0.287	0.973	(0.961, 0.986)	**0.032**

Effects are estimated from a GEE empirical estimator.

Table S5. *HP2* additive effect on NCI in the AFR and EUR participants with adjustment of CSF LLQ status over time.

	AFR (n=393, Rec.=1,358)			EUR (n=371, Rec.=1,461)		
Variable	OR	95% CI	*p*_value	OR	95% CI	*p*_value
Intercept	1.624	(0.574, 4.601)	0.641	0.024	(0.009, 0.061)	$\mathbf{7.370 \times 10^{-5}}$
HP2 Additive	0.258	(0.097, 0.690)	0.168	3.962	(2.102, 7.467)	**0.030**
Age	0.963	(0.942, 0.984)	0.089	1.060	(1.040, 1.081)	**0.003**
>LLQ	1.083	(0.914, 1.284)	0.637	1.328	(1.156, 1.527)	**0.041**
Sex: Female	1.181	(0.925, 1.507)	0.497	1.074	(0.754, 1.529)	0.841
CD4+ Nadir (Cells/μL)	1.000	(0.999, 1.000)	0.583	0.999	(0.998, 0.999)	**0.018**
Comorbidity: Mild-Moderate	2.373	(1.886, 2.985)	$\mathbf{1.653 \times 10^{-4}}$	3.557	(2.833, 4.467)	$\mathbf{2.525 \times 10^{-8}}$
HP2 Additive × Age	1.025	(1.003, 1.048)	0.255	0.976	(0.963, 0.989)	0.065

Effects are estimated from a GEE empirical estimator.

Table S6. *HP2* dominant effect on learning domain impairment in AFR and EUR participants over time.

	AFR (n=392, Rec.=1,347)			EUR (n=371, Rec.=1,449)		
Variable	OR	95% CI	p_value	OR	95% CI	p_value
Intercept	0.715	(0.285, 1.792)	0.715	0.018	(0.006, 0.050)	$\mathbf{1.169 \times 10^{-4}}$
HP2 Dominant	0.652	(0.223, 1.908)	0.690	14.526	(4.637, 45.502)	**0.019**
Age	0.983	(0.965, 1.002)	0.364	1.067	(1.046, 1.089)	**0.001**
Sex: Female	1.281	(1.037, 1.582)	0.241	0.716	(0.528, 0.969)	0.270
Log_{10}(Plasma HIV RNA)	1.014	(0.959, 1.073)	0.802	0.986	(0.932, 1.044)	0.805
CD4+ Nadir (Cells/μL)	1.000	(1.000, 1.001)	0.548	0.999	(0.999, 1.000)	0.135
Comorbidity: Mild-Moderate	1.789	(1.470, 2.178)	**0.003**	2.397	(1.971, 2.916)	$\mathbf{7.905 \times 10^{-6}}$
HP2 Dominant × Age	1.000	(0.977, 1.024)	0.988	0.951	(0.929, 0.973)	**0.030**

Effects are estimated from a GEE empirical estimator.

Table S7. *HP2* additive effect on verbal domain impairment in AFR and EUR participants over time.

	AFR (n=392, Rec.=1,347)			EUR (n=371, Rec.=1,449)		
Variable	OR	95% CI	p_value	OR	95% CI	p_value
Intercept	0.238	(0.057, 0.996)	0.316	0.655	(0.263, 1.630)	0.642
HP2 Additive	0.194	(0.062, 0.602)	0.148	0.259	(0.146, 0.461)	**0.019**
Age	0.985	(0.958, 1.013)	0.597	0.970	(0.953, 0.988)	0.092
Sex: Female	0.748	(0.511, 1.096)	0.447	1.833	(1.330, 2.526)	0.059
Log_{10}(Plasma HIV RNA)	1.108	(0.979, 1.255)	0.407	1.031	(0.956, 1.111)	0.689
CD4+ Nadir (Cells/μL)	1.000	(0.999, 1.001)	0.719	1.000	(0.999, 1.001)	0.844
Comorbidity: Mild-Moderate	1.326	(0.966, 1.821)	0.373	1.923	(1.513, 2.443)	**0.006**
HP2 Additive × Age	1.025	(1.001, 1.051)	0.305	1.029	(1.016, 1.042)	**0.023**

Effects are estimated from a GEE empirical estimator.

Table S8. *HP2* dominant effect on speed of information processing domain impairment in AFR and EUR participants over time.

Variable	AFR (n=392, Rec.=1,347)			EUR (n=371, Rec.=1,449)		
	OR	95% CI	p_value	OR	95% CI	p_value
Intercept	1.755	(0.525, 5.866)	0.641	0.045	(0.014, 0.141)	**0.007**
HP2 Dominant	0.038	(0.009, 0.168)	**0.027**	1.583	(0.438, 5.719)	0.721
Age	0.939	(0.914, 0.965)	**0.021**	1.023	(1.002, 1.044)	0.279
Sex: Female	1.252	(0.928, 1.687)	0.452	1.245	(0.870, 1.781)	0.542
Log_{10}(Plasma HIV RNA)	0.994	(0.909, 1.086)	0.942	1.035	(0.939, 1.141)	0.726
CD4+ Nadir (Cells/μL)	0.999	(0.998, 1.000)	0.248	0.999	(0.998, 0.999)	0.058
Comorbidity: Mild-Moderate	1.946	(1.462, 2.590)	**0.020**	2.969	(2.317, 3.805)	$\mathbf{1.152 \times 10^{-5}}$
HP2 Dominant × Age	1.072	(1.037, 1.108)	**0.038**	0.997	(0.972, 1.023)	0.906

Effects are estimated from a GEE empirical estimator.

Table S9. *HP* effects on cognitive domain T-scores in the AFR participants (n=392, Rec.=1,374) over time.

Verbal T-score				Memory T-score			
Variable	Estimate	Std. Error	*p*_value	Variable	Estimate	Std. Error	*p*_value
Intercept	45.700	4.093	$\mathbf{<2 \times 10^{-16}}$	Intercept	51.842	4.421	$\mathbf{<2 \times 10^{-16}}$
HP2 Dominant	10.499	4.942	**0.034**	*HP1* Dominant	−9.855	4.995	**0.049**
Age	0.120	0.087	0.170	Age	−0.073	0.096	0.449
Sex: Female	0.837	0.835	0.316	Sex: Female	−0.863	0.846	0.308
Log_{10}(Plasma HIV RNA)	−0.240	0.167	0.150	Log_{10}(Plasma HIV RNA)	−0.037	0.220	0.867
CD4+ Nadir (Cells/μL)	−0.001	0.003	0.683	CD4+ Nadir (Cells/μL)	5.450×10^{-4}	0.003	0.834
Comorbidity: Mild-Moderate	−1.100	0.834	0.187	Comorbidity: Mild-Moderate	−3.037	0.812	$\mathbf{1.858 \times 10^{-4}}$
HP2 Dominant × Age	−0.196	0.108	0.071	*HP1* Dominant × Age	0.203	0.111	0.066

Effects are estimated from a GEE empirical estimator.

Table S10. *HP2* dominant effect on domain T-scores in the EUR participants (n=371, Rec.=1,449) over time.

Variable	Learning T-score			Memory T-score			Motor T-score		
	Estimate	Std. Error	p_value	Estimate	Std. Error	p_value	Estimate	Std. Error	*p*_value
Intercept	55.202	3.783	$\mathbf{<2 \times 10^{-16}}$	62.734	3.765	$\mathbf{<2 \times 10^{-16}}$	61.417	5.596	$\mathbf{<2 \times 10^{-16}}$
HP2 Dominant	−10.956	4.068	**0.007**	−11.080	4.079	**0.007**	−11.850	6.043	**0.050**
Age	−0.199	0.074	**0.007**	−0.342	0.075	$\mathbf{5.749 \times 10^{-6}}$	−0.276	0.115	**0.016**
Sex: Female	−0.456	1.288	0.723	−0.681	1.321	0.606	−2.005	1.870	0.283
Log_{10}(Plasma HIV RNA)	−0.161	0.255	0.529	0.042	0.317	0.894	0.027	0.229	0.905
CD4+ Nadir (Cells/μL)	9.881×10^{-4}	0.003	0.731	0.006	0.003	0.097	0.002	0.003	0.521
Comorbidity: Mild-Moderate	−4.187	0.925	$\mathbf{5.967 \times 10^{-6}}$	−3.800	0.954	$\mathbf{6.801 \times 10^{-5}}$	−5.055	1.138	$\mathbf{8.958 \times 10^{-6}}$
HP2 Dominant × Age	0.213	0.088	**0.016**	0.212	0.091	**0.020**	0.187	0.129	0.147

Effects are estimated from a GEE empirical estimator.

Table S11. Baseline *HP2* additive effect on NCI in the AFR and EUR participants.

Variable	AFR			EUR		
	Estimate	Std. Error	*p*_value	Estimate	Std. Error	*p*_value
Intercept	-0.458	0.848	0.589	-1.202	0.902	0.183
HP2 Dosage	-0.403	0.165	0.015	0.491	0.173	0.004
Log_{10}(Plasma HIV RNA)	-0.018	0.089	0.839	0.013	0.096	0.889
Comorbidity: Mild-Moderate	0.673	0.227	0.003	1.246	0.246	4.25×10^{-7}
Age (Month)	-0.011	0.015	0.463	-0.008	0.014	0.552
Sex: Female	0.166	0.239	0.487	0.300	0.350	0.390
CD4+ Nadir (Cells/μL)	-3.08×10^{-4}	6.36×10^{-4}	0.628	-2.35×10^{-3}	7.53×10^{-4}	0.002

Effect estimates are from a logistic regression model.

Table S12. Baseline *HP1* dominant effect on NCI in the EUR participants.

Variable	Estimate	Std. Error	p_value
Intercept	-0.158	0.859	0.854
HP1 Dominant	-0.612	0.237	0.010
Log_{10}(Plasma HIV RNA)	2.54e-4	0.095	0.998
Comorbidity: Mild-Moderate	1.240	0.246	4.48×10^{-7}
Age (Month)	-0.009	0.014	0.510
Sex: Female	0.309	0.350	0.379
CD4+ Nadir (Cells/μL)	-2.32×10^{-3}	7.47×10^{-4}	1.87×10^{-3}

Effect estimates are from a logistic regression model.

Table S13. *HP* effects on CSF HIV RNA in the AFR participants over time.

AFR (n=326, Rec.=1,125)							
Variable	Estimate	Std. Error	p_value	Variable	Estimate	Std. Error	p_value
Intercept	0.891	0.184	$\mathbf{1.235 \times 10^{-6}}$	Intercept	0.430	0.176	**0.015**
HP2 Additive	−0.179	0.082	**0.029**	*HP1* Dominant	0.392	0.129	**0.002**
Log_{10}(Plasma HIV RNA)	0.364	0.047	$\mathbf{9.659 \times 10^{-15}}$	Log_{10}(Plasma HIV RNA)	0.577	0.039	$\mathbf{<2 \times 10^{-16}}$
Age	0.004	0.003	0.148	Age	0.004	0.003	0.170
HP2 Additive × Log_{10}(Plasma HIV RNA)	0.085	0.035	**0.014**	*HP1* Dominant × Log_{10}(Plasma HIV RNA)	−0.175	0.051	$\mathbf{6.479 \times 10^{-4}}$

Effects are estimated from a GEE empirical estimator.

Table S14. *HP2* effects on NCI in the EUR participants over time with adjustment of plasma HIV RNA and HIV duration (n=370, Rec.=1,450).

Variable	OR	95% CI	p_value	Variable	OR	95% CI	p_value
Intercept	0.021	(0.008, 0.058)	$\mathbf{1.522 \times 10^{-4}}$	Intercept	0.019	(0.007, 0.049)	$\mathbf{2.327 \times 10^{-5}}$
HP2 Dominant	9.710	(3.223, 29.257)	**0.039**	*HP2* Additive	4.906	(2.716, 8.864)	**0.007**
Age	1.043	(1.023, 1.063)	**0.028**	Age	1.047	(1.028, 1.067)	**0.015**
Sex: Female	1.174	(0.859, 1.605)	0.608	Sex: Female	1.204	(0.873, 1.660)	0.564
Comorbidity: Mild-Moderate	3.124	(2.525, 3.865)	$\mathbf{8.895 \times 10^{-8}}$	Comorbidity: Mild-Moderate	3.143	(2.538, 3.892)	$\mathbf{8.436 \times 10^{-8}}$
Log_{10}(Plasma HIV RNA)	1.246	(1.117, 1.389)	**0.044**	Log_{10}(CSF HIV RNA)	1.243	(1.114, 1.387)	**0.047**
HIV Duration (Month)	1.004	(1.002, 1.006)	**0.037**	HIV Duration (Month)	1.004	(1.002, 1.006)	**0.039**
CD4+ Nadir (Cells/μL)	0.998	(0.998, 0.999)	**0.007**	CD4+ Nadir (Cells/μL)	0.998	(0.998, 0.999)	**0.008**
HP2 Dominant × Age	0.962	(0.941, 0.984)	0.083	*HP2* Additive × Age	0.971	(0.959, 0.983)	**0.019**
HIV Duration (Month) × Log_{10}(Plasma HIV RNA)	0.999	(0.999, 1.000)	0.189	HIV Duration (Month) × Log_{10}(CSF HIV RNA)	0.999	(0.999, 1.000)	0.204

Effects are estimated from a GEE empirical estimator.

Table S15. *HP2* effects on NCI in the EUR participants over time with adjustment of CSF HIV RNA and HIV duration (n=318, Rec.=1,274).

Variable	OR	95% CI	p_value	Variable	OR	95% CI	p_value
Intercept	0.015	(0.005, 0.042)	$\mathbf{4.936 \times 10^{-5}}$	Intercept	0.010	(0.003, 0.027)	$\mathbf{7.192 \times 10^{-6}}$
HP2 Dominant	4.390	(1.437, 13.411)	0.185	*HP2* Additive	3.828	(2.049, 7.153)	**0.032**
Age	1.039	(1.020, 1.059)	**0.042**	Age	1.050	(1.029, 1.071)	**0.014**
Sex: Female	1.085	(0.772, 1.526)	0.811	Sex: Female	1.105	(0.780, 1.565)	0.774
Comorbidity: Mild-Moderate	3.591	(2.850, 4.523)	$\mathbf{3.071 \times 10^{-8}}$	Comorbidity: Mild-Moderate	3.570	(2.832, 4.500)	$\mathbf{3.949 \times 10^{-8}}$
Log_{10}(Plasma HIV RNA)	1.610	(1.359, 1.907)	**0.005**	Log_{10}(CSF HIV RNA)	1.594	(1.342, 1.895)	**0.007**
HIV Duration (Month)	1.007	(1.005, 1.010)	$\mathbf{5.607 \times 10^{-4}}$	HIV Duration (Month)	1.007	(1.005, 1.010)	$\mathbf{7.954 \times 10^{-4}}$
CD4+ Nadir (Cells/μL)	0.999	(0.998, 0.999)	**0.029**	CD4+ Nadir (Cells/μL)	0.999	(0.998, 0.999)	**0.034**
HP2 Dominant × Age	0.977	(0.955, 0.999)	0.305	*HP2* Additive × Age	0.976	(0.964, 0.989)	0.067
HIV Duration (Month) × Log_{10}(Plasma HIV RNA)	0.998	(0.997, 0.999)	**0.004**	HIV Duration (Month) × Log_{10}(CSF HIV RNA)	0.998	(0.997, 0.999)	**0.006**

Effects are estimated from a GEE empirical estimator.

3 SUPPLEMENTAL FIGURES

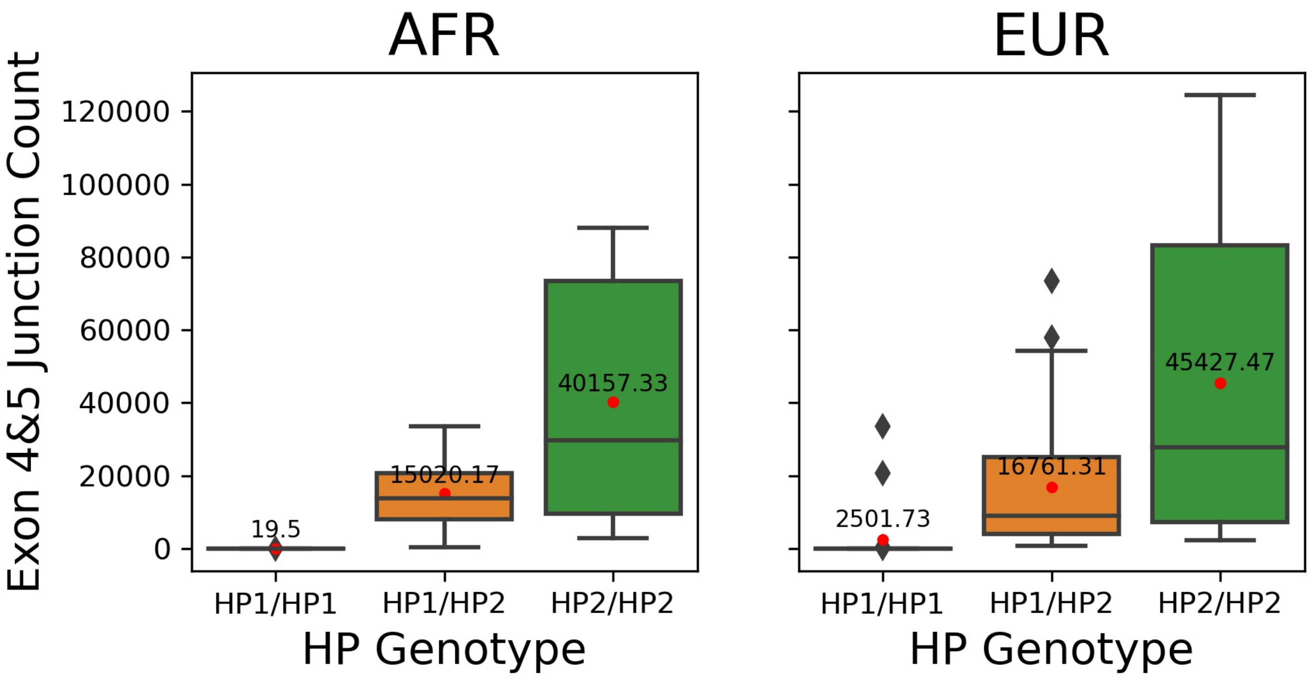

Figure S1. Distribution of junction 45 across HP genotypes in GTEx data. Boxplot showing the distribution of the junction between *HP* exon4 and exon5 vs. imputed *HP* genotypes in AFR and EUR GTEx data.

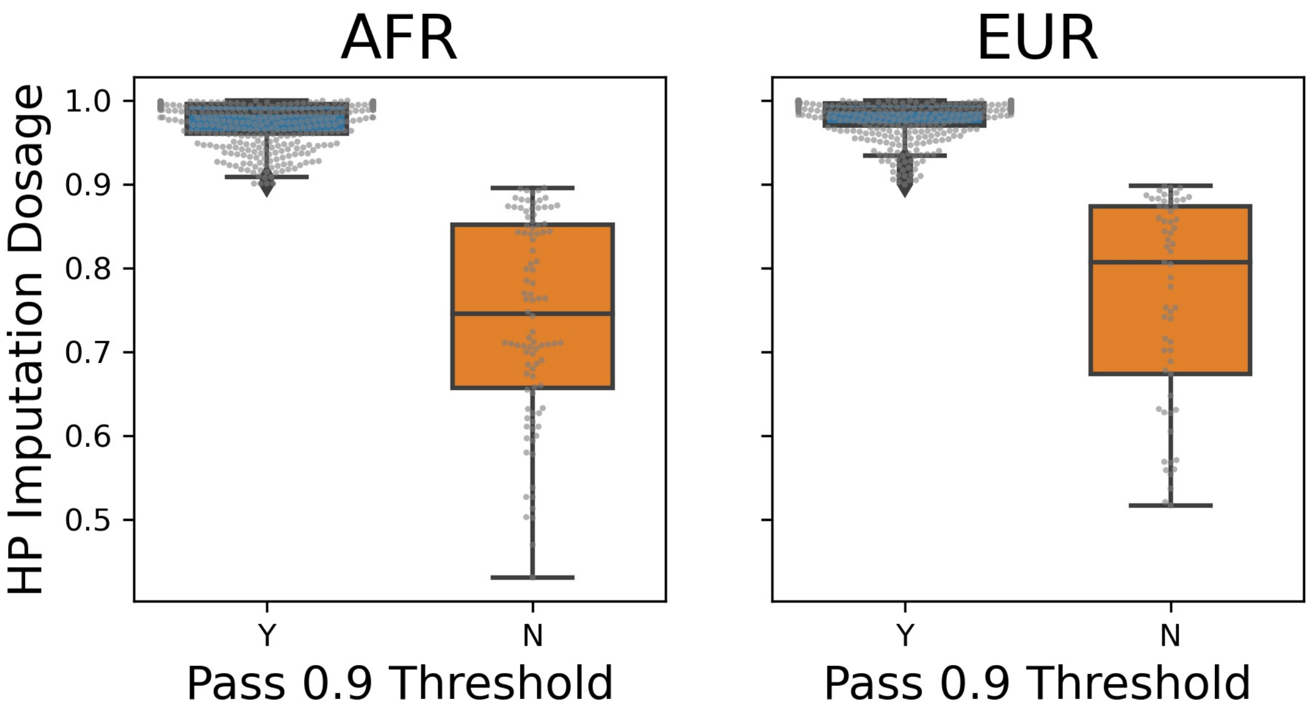

Figure S2. HP imputation dosages. Boxplot showing the distribution of *HP* imputation dosages that passed and didn't pass the 0.9 hardcall threshold in AFR and EUR.

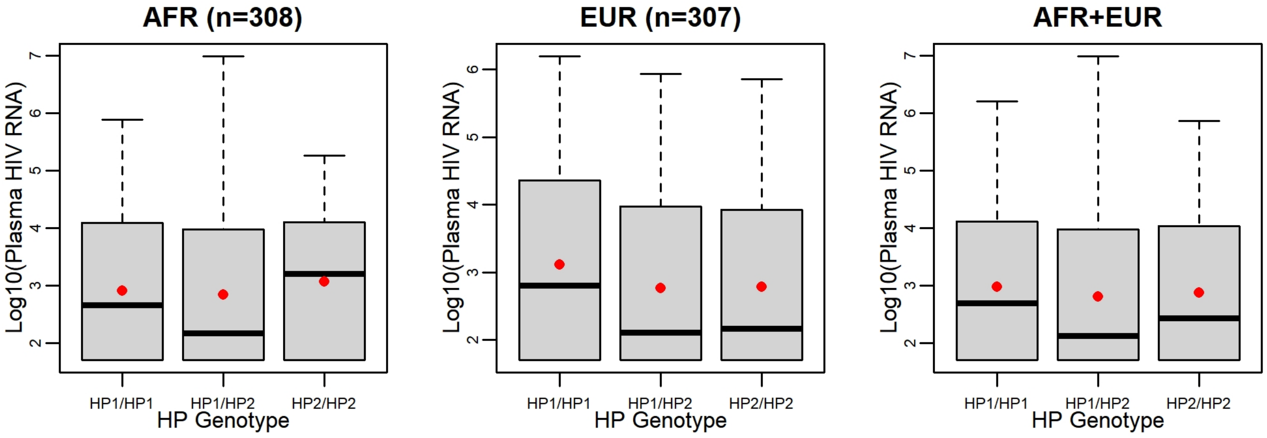

Figure S3. Baseline Plasma HIV RNA vs. *HP* genotypes in log base 10 scale. In each box, the black horizontal bar indicates the median and the red dot shows the mean value.

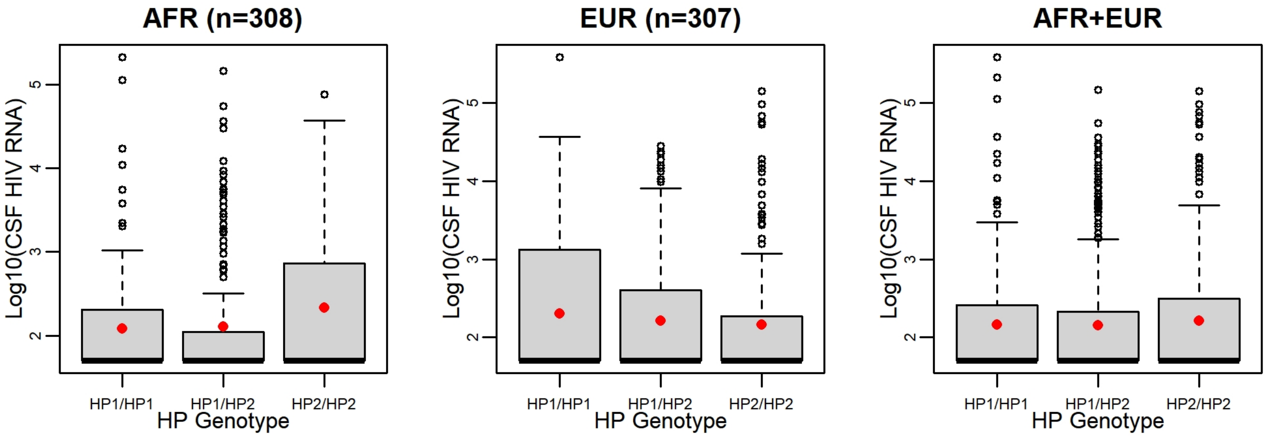

Figure S4. Baseline CSF HIV RNA vs. *HP* genotypes. In each box, the black horizontal bar indicates the median and the red dot shows the mean value.

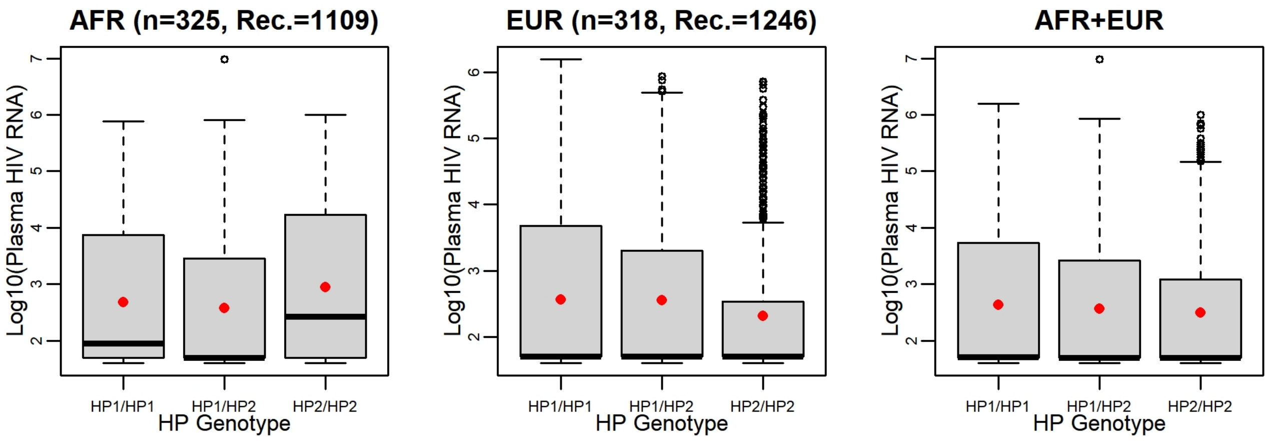

Figure S5. Plasma HIV RNA vs. *HP* genotypes with repeated measurements over age. In each box, the black horizontal bar indicates the median and the red dot shows the mean value.

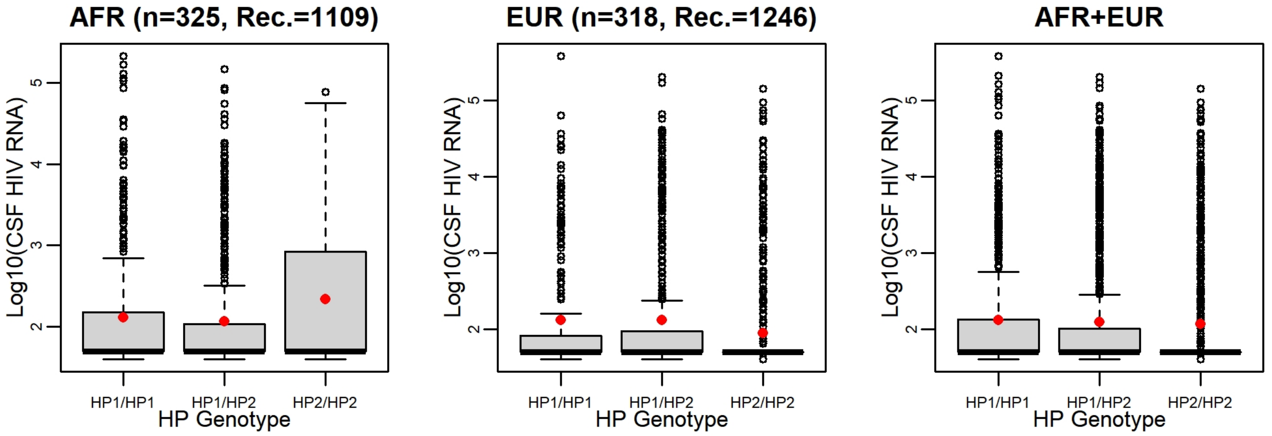

Figure S6. CSF HIV RNA vs. *HP* genotypes with repeated measurements over age. In each box, the black horizontal bar indicates the median and the red dot shows the mean value.
